# Activity-regulated cytoskeletal-associated protein (Arc) in presynaptic terminals and extracellular vesicles in hippocampal synapses

**DOI:** 10.3389/fnmol.2023.1225533

**Published:** 2023-11-06

**Authors:** Håvard Ringsevjen, Daniel Lawer Egbenya, Malte Bieler, Svend Davanger, Suleman Hussain

**Affiliations:** ^1^Division of Anatomy, Department of Molecular Medicine, Institute of Basic Medical Sciences, University of Oslo, Oslo, Norway; ^2^Department of Physiology, School of Medical Sciences, College of Health and Allied Sciences, University of Cape Coast, Cape Coast, Ghana; ^3^Institute of Oral Biology, Faculty of Dentistry, University of Oslo, Oslo, Norway

**Keywords:** Arc, hippocampus, synapse, extracellular vesicles, electron microscopy

## Abstract

The activity-regulated cytoskeleton-associated protein (Arc/Arg3.1) is a neuron-specific immediate early gene (IEG) product. The protein regulates synaptic strength through modulation of spine density and morphology, AMPA receptor endocytosis, and as being part of a retrovirus-like inter-cellular communication mechanism. However, little is known about the detailed subsynaptic localization of the protein, and especially its possible presynaptic localization. In the present study, we provide novel electron microscopical data of Arc localization at hippocampal Schaffer collateral synapses in the CA1 region. The protein was found in both pre-and postsynaptic cytoplasm in a majority of synapses, associated with small vesicles. We also observed multivesicular body-like structures positive for Arc. Furthermore, the protein was located over the presynaptic active zone and the postsynaptic density. The relative concentration of Arc was 25% higher in the postsynaptic spine than in the presynaptic terminal. Notably, small extracellular vesicles labeled for Arc were detected in the synaptic cleft or close to the synapse, supporting a possible transsynaptic transmission of the protein in the brain.

## Introduction

The activity-regulated cytoskeleton-associated protein (Arc/Arg3.1) was discovered independently by two labs in 1995 and is recognized as a member of the immediate-early gene (IEG) family (Link et al., 1995; Lyford et al., 1995). Its expression was found to be upregulated following synaptic activity, such as after induction of convulsive seizures or high-frequency stimulation resulting in long-term potentiation (LTP) (Link et al., 1995; Szyndler et al., 2013).

The Arc gene encodes a protein of 45 kDa that is associated with the actin cytoskeleton (Lyford et al., 1995). The protein has no identifiable family members or biochemically defined domains, which makes it difficult to understand its molecular interactions and functional roles.

Arc is reported to have several functions during synaptic plasticity (Chowdhury et al., 2006; Bramham et al., 2010; Korb and Finkbeiner, 2011; Shepherd and Bear, 2011). It is involved in endocytosis of glutamate receptors of the AMPA type (AMPAR), by interaction with endophilin and dynamin (Chowdhury et al., 2006). Arc is also found to be involved in regulating spine size and spine density (Peebles et al., 2010). Specifically, over-expression of Arc in hippocampal neurons has been shown to increase the spine density *in vitro*, as well as increase the proportion of thin spines (Peebles et al., 2010). Thus, Arc is involved in both functional and structural aspects of synaptic plasticity.

In mammalian CNS synapses, Arc has been localized in the postsynaptic density (PSD), where the PSD-95 protein has been reported as the most abundant Arc-interacting protein (Fernandez et al., 2017). It is also highly expressed in dendrites and the nucleus, while expression in presynaptic compartments is not yet well established (Husi et al., 2000; Irie et al., 2000; Fujimoto et al., 2004; Moga et al., 2004; Rodriguez et al., 2005; Bloomer et al., 2007).

Interestingly, Arc contains Group-specific antigen (Gag)-like amino acid sequences (Campillos et al., 2006) that are typically found in retroviruses such as HIV. This led to the hypothesis that Arc also could behave like a virus. In fact, recent research indicates that Arc may contribute to intercellular signaling in the nervous system through extracellular vesicles (EVs), and that the Gag proteins multimerize into capsids, which can bind and package RNA (Ashley et al., 2018).

In both physiological and pathological conditions, exosomes and other extracellular vesicles have been found to be involved in the phenomenon of trans-cellular communication (Basso and Bonetto, 2016). For instance, Basso and Bonetto (2016) have reported that the spread of some neurodegenerative diseases, such as ALS, may be mediated by the transfer of proteins via exosomes across cells. Similarly, at the *Drosophila* neuromuscular junction, dArc1 protein forms capsid-like structures in the neuron and binds dArc1 mRNA before being loaded into extracellular vesicles and transferred from presynaptic boutons to postsynaptic muscles in a transsynaptic transfer mechanism (Ashley et al., 2018).

Endogenous Arc has been showed to be released by cultured Drosophila neurons in extracellular vesicles (Pastuzyn et al., 2018). Such extracellular vesicles, or exosomes, may be involved in trans-synaptic transmission of proteins (Wang et al., 2017), because after their release from the neuronal presynaptic terminal, they can be taken up by postsynaptic sites at *Drosophila* neuromuscular junction (Korkut et al., 2013). Exosomes are membrane-derived nanovesicles (30–100 nm) which are produced in multivesicular bodies and are exocytosed by cells into the extracellular space (Thery et al., 2002; Conde-Vancells et al., 2008; Lakkaraju and Rodriguez-Boulan, 2008; Simons and Raposo, 2009). This occurs when portions of the multivesicular bodies fuse with the plasma membrane. This vesicular trans-synaptic communication, however, has so far only been demonstrated in the Drosophila neuromuscular junction. In the CNS, the proposed vehicle for such trans-synaptic communication, the Arc-containing extracellular vesicle, has not yet been observed. Another missing component in the picture of Arc in central synapses is that the possible presence of Arc in presynaptic terminals is not yet sufficiently characterized.

We now report the presence of Arc-immunopositive multivesicular body-like structures (MVBLS), its presence in both presynaptic and postsynaptic cytoplasm, as well as Arc-immunopositive extracellular vesicles (EVs) in the synaptic cleft of glutamatergic synapses in the rat hippocampus.

## Materials and methods

### Antibodies

Primary antibody: The polyclonal rabbit Arc antibody (Synaptic Systems, Göttingen, Germany, Cat#156003, RRID: AB_887694) was raised against recombinant protein corresponding to AA 1–396 from mouse Arc (UniProt Id: Q9WV31), used at 1:400 for electron microscopy (EM), 1:500 for light microscopy (LM) and 1:600 for western blot (WB). The specificity of this Arc antibody has been verified by immunocytochemistry of dissociated hippocampal neuron cultures prepared from wild type (WT) and Arc knockout (KO) littermates (Niere et al., 2012), and has been characterized in our lab previously (Egbenya et al., 2023). Monoclonal actin antibody (Millipore, Germany, Cat# MAB1501, RRID:AB_2223041), raised against amino acids 357–375 at the C-terminus of actin of human origin, used at 1:20000 for WB.

Secondary antibodies: Goat anti-rabbit coupled to 10 nm gold particle (Abcam, Cambridge, United Kingdom, IgG H&L antibody, Cat#AB27234, Lot#GR73433 and Lot#GR104536-1, RRID: AB_954427) used at 1:40 for EM. Biotinylated donkey anti-rabbit antibody (GE healthcare, United Kingdom, lot#9554939) was used at 1:100 for LM. For WB, a monoclonal anti-rabbit IgG alkaline phosphatase antibody produced in mouse (Sigma, United States, lot#083M4782V) was used at 1:10,000.

### Animals

For the WB experiments, three Wistar rats weighing 250–300 g (Scanbur, Nittedal, Norway) were used for the regional brain experiments. Three Wistar rats weighing 250–300 g (Scanbur, Nittedal, Norway) were used for the LM experiments. Four Sprague–Dawley rats (Harlan Sprague–Dawley Inc., Indiana, United States) weighing 120–240 g were used for the EM experiments. Experimental protocols were approved by the Institutional Animal Care and Use Committee and conform to the National Institute of Health guidelines for the care and use of animals, as well as international laws on protection of laboratory animals, with the approval of a local bioethical committee and under the supervision of a veterinary commission for animal care and comfort. The animals were treated in accordance with the guidelines of the Norwegian Committee on Animal Experimentation (Norwegian Animal Welfare Act and European Communities Council, Directive of 24 November 1986-86/609/EEC). Every effort was made to minimize the number of animals used and their sufferings. This article does not contain any studies with human participants performed by any of the authors.

### Light microscopy

Whole rat brain vibratome sections (50 μm) from three rats were immunostained with anti-Arc antibody using the labeled streptavidin-biotin method to optimize antibody concentration and labeling conditions. The sections were incubated in 1 M ethanolamine in sodium phosphate buffer (0.1 M, pH 7.4) for 30 min. After washing 3 × 1 min in sodium phosphate buffer, the sections were incubated in blocking buffer (10% normal goat serum in sodium phosphate buffer) for 1 h prior to primary antibody incubation overnight (diluted in blocking buffer at concentration 1:100). The following day, the sections were rinsed in sodium phosphate buffer for 3 × 5 min, and then incubated in 1% normal goat serum in sodium phosphate buffer for 20 min. The sections were incubated in biotinylated secondary antibody diluted in the same buffer for 1 h at room temperature. Washing 3 × 5 min with sodium phosphate buffer followed. Then the sections were incubated with streptavidin-biotinylated horse-radish peroxidase (HRP) diluted in sodium phosphate buffer with 1% normal goat serum for 1 h, before washing in sodium phosphate buffer for 5 × 10 min. As the final steps, the sections were incubated in 0.05% diaminobenzidine (DAB) in sodium phosphate buffer for 5 min, and then incubated in 0.01% H_2_O_2_ and 0.05% DAB diluted in buffer for 6 min, before final washing in sodium phosphate buffer 3 × 5 min. Sections were mounted on glass slides with glycerol gelatin.

### Western blot

Three rats were decapitated and the brains were removed. Brain regions of interest were dissected and submerged in ice-cold Hepes-buffered sucrose (0,32 M sucrose, 4 mM Hepes, pH 7.4) containing protease and phosphatase inhibitor cocktails. Corresponding brain regions from the different rats were combined and homogenized in a Hepes buffer with a glass-Teflon homogenizer (10–15 strokes) and centrifuged (800–1,000 g, 10 min, 4°C). 20 μg of protein extracts (brain region materials) were run on a 4–20% SDS-acrylamide gel (Bio-Rad Laboratories Inc., United States, Cat# 345-0007) at 200 V and 400 mA for 50–55 min followed by blotting for 30 min at 25 V, 1 A. The different protein extract amounts were used to optimize band intensities for quantification. Prior to this, the samples were mixed with loading buffer (62.5 mM Tris–HCl, 10% glycerol, 2% SDS, 5% 2-mercaptoethanol, 0.025% bromophenol blue). The membrane was blocked with 5% skim-milk solution for 60 min followed by an overnight incubation with primary antibody (in 2.5% skim-milk solution). Secondary antibody incubation (in 1.25% skim-milk solution), washing (4 × 15 min using TBS-T buffer) and signal detection using ECF substrate (GE Healthcare, United Kingdom, Cat#1067873) were performed. BioRad Scanner (Bio-Rad Laboratories, Inc., United States) was used to visualize the fluorescence signals. Three blots were used for the semiquantitative WB analyses. We utilized photoshop to create a rectangular marker designed to tightly enclose the bands on the WB membrane images. Values “mean” and “pixels” were then transferred to an Excel sheet. The intensity of each band was calculated by taking the mean times the pixels divided by 1,000. The background was measured and substracted from mean band intensity. Band intensity of the blot was normalized to loading controls (actin). The regional WB were performed to visualize brain regional differences in expression of Arc, not for statistical comparison.

### Perfusion fixation of rats

Four rats were used for EM. We optimized conditions for ultrastructural visualization of vesicles with two different fixation protocols. Two of the rats were deeply anaesthetized with Equithesin (0.4 mL/100 g, i.p.) and transcardially perfused with a flush of 30 mL of 2% Dextran-T70 in 0.1 M phosphate buffer and thereafter with 1.0 L of a fixative made up of glutaraldehyde (0.5%) and formaldehyde (4.0%) in the same buffer. Two of the other rats were deeply anesthetized with an i.p. injection of a mixture of ketamine (100–200 mg/kg) and xylaxine (10–20 mg/kg) and sacrificed by transcardial perfusion with 30 mL 4% Dextran-T70 in 0.1 M sodium phosphate buffer (pH 7.4) followed by a mixture of 0.1% glutaraldehyde and 4.0% formaldehyde in the same buffer. The fixed brains were left *in situ* overnight at 4°C, and then dissected out from the skulls and stored in the above-mentioned fixatives after diluting 1: 10 in 0.1 M phosphate buffer at 4°C. No differences between the two protocols were observed, so all four brains were used in the study.

### Postembedding immunocytochemistry

Small (0.5–1.0 mm) blocks dissected from the CA1 area of the hippocampus were freeze-substituted, sectioned, and immunolabeled as described previously (Hussain et al., 2019). After sectioning and mounting on nickel grids, the sections were first incubated in TBST (20 mL 0.05 M Tris–HCl, 180 mL dH2O with 1.62 g NaCl, 0.02 g Triton X-100) with glycine for 10 min. Then they were incubated in blocking buffer (2% human serum albumin in TBST) for 10 min, before incubation overnight in primary antibody diluted in blocking buffer. Next day, the sections were rinsed 3 × 10 min in TBST, before incubating in blocking buffer for 10 min. They were then incubated for 2 h in secondary antibody coupled to 10 nm colloidal gold in blocking buffer. Subsequently, the grids were briefly washed with distilled water six times. After drying, they were incubated in drops of 1% uranyl acetate for 1.5 min, then washed three times with distilled water, and incubated in drops of 0.3% lead citrate for 1.5 min, and finally washed with distilled water and dried.

### Electron microscopy and quantification

Four rats were used for the quantitative postembedding electron microscopy. From each animal, three ultrathin sections were immunolabeled. Twenty-five synaptic profiles from each section were selected for the quantitative analysis. A total of 291 synaptic profiles were quantified. From each image of a synapse, we examined 4 regions of interest (ROIs). Similar selections were made for the myelin profiles in separate images. Five myelin profiles from each section were selected for the quantitative analysis. A total of 56 images of myelin were used for the quantification. Electron micrographs were obtained at random from the middle layer of the stratum radiatum of the dorsal CA1 region of the hippocampus, midway between the stratum radiatum and stratum lacunosum. Asymmetric synapses in this location are well-defined glutamatergic synapses. Immunolabeling was quantified as number of gold particles/μm of membrane length in asymmetric synapses (from Schaffer collaterals) and as number of gold particles/μm^2^ in the intracellular compartments. Specific plasma membrane and cytoplasmic compartments were defined as ROIs and used for quantification. These included the presynaptic cytoplasm (PreCy), the active zone (AZ), the postsynaptic cytoplasm (PoCy) and the postsynaptic density (PSD). Images of synaptic profiles clearly showing the ROI were acquired for the quantitative analysis. The following total numbers of synaptic profiles were analyzed for the different ROI: PreCy = 291, PoCy = 291, AZ = 291, PSD = 291. Arc immunonegative synaptic profiles were also included in the quantitative analyses. For the quantitative analysis of percentage of synapses exhibiting MVBSL, EVs, and Arc immunolabeling in different synaptic compartments, we utilized a set of 190 synaptic profiles with well-preserved ultrastructure. Excitatory synapses were identified by the presence of two closely aligned membranes with a synaptic cleft, a prominent postsynaptic density and circular synaptic oriented vesicles at the presynaptic side. An in-house extension to analysis connected with SPSS (SPSS Inc., Chicago, IL, United States) was used to evaluate the gold particle labeling of the specific plasma membrane and cytoplasmic compartments. The software calculated area particle density (number per unit area) over cytoplasmic compartments and linear particle density (number per unit length of curve) over membrane domains. In the latter case, it measured the distance from each particle-center to the membrane and included only those particles which were within an operator-defined distance from the curve segment. For general plasma membranes, the inclusion distance was symmetric between −21 nm and + 21 nm (negative value signifying an intracellular location). The inclusion distance was defined as the distance between the epitope and the center of the gold particle, corresponding to the radius of the particle (5 nm) and the length of the interposed primary antibody (8 nm) and length of the secondary antibody (8 nm). Data for particles were collected in ASCII files as flat tables and exported to SPSS for further statistical and graphical analysis.

### Data analysis and statistics

Quantitative data from EM were analyzed with SPSS by performing a Mann–Whitney non-parametric test, as all criteria for this test were met and the data were not normally distributed. The criterion for statistical significance was set to *p* < 0.05 as standard.

## Results

### Arc is abundant in the hippocampus and brain cortex

**Figure 1:**
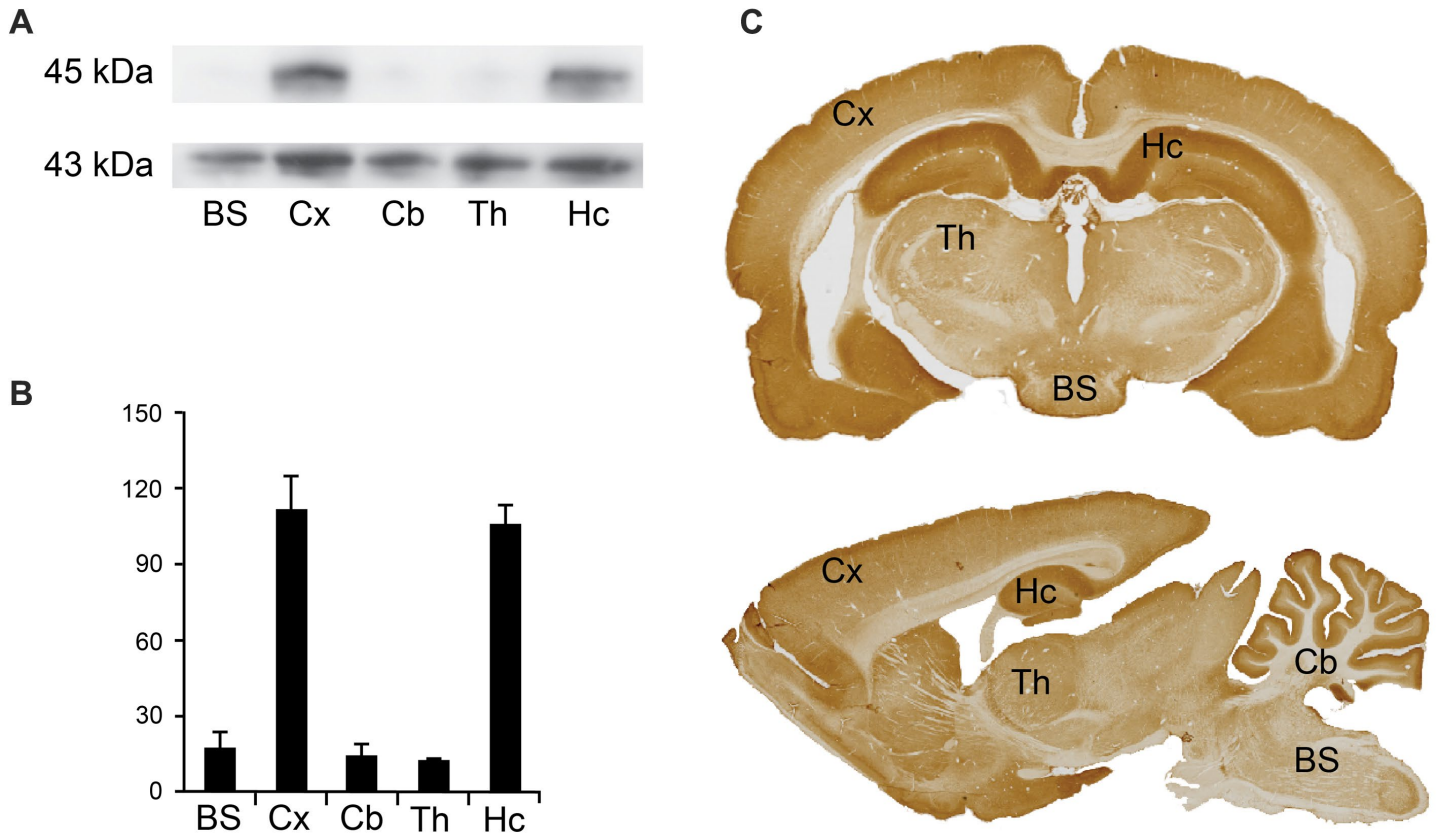
Regional Arc expression in the brain. **(A)** Western blots from rat brain homogenate from different brain regions, with antibodies against Arc (upper lane) and actin as a loading control (lower lane) showing bands at 45 kDa and 43 kDa, respectively. BS: brain stem; Cx: cortex; Cb: cerebellum; Th: thalamus; Hc: hippocampus. **(B)** Quantification of mean band intensities, indicating highest concentration of Arc in the cortex and the hippocampus. Values are recorded as mean ± SEM. The blots and graphs are based on three technical replicates from one homogenate made from regional brain tissue from three animals. **(C)** Light micrographs of rat brain sections immunolabeled for Arc. BS, brain stem; Cx, cortex; Cb, cerebellum; Hc, hippocampus.

To determine the expression of Arc in different brain regions in the central nervous system (CNS), we performed western blotting and immunostaining for light microscopy. The anti-Arc antibody gave a single immunoreactive band at the expected molecular weight. Arc was expressed in different brain regions (Figure 1), including the brain stem, cortex, cerebellum, thalamus, and the hippocampus. The highest band intensities of Arc, normalized against actin, were observed in the cortex and the hippocampus (Figures 1A,B). Concentrations seem to be lower in the brain stem, thalamus and the cerebellum (Figures 1A,B). These immunoblotting results correspond to the immunoreactivity observed for immunostained sections using the labeled streptavidin-biotin method for light microscopy (Figure 1C). Most prominent labeling intensities were observed in the cortex and the hippocampus (Figure 1C). While the cerebellar cortex showed prominent immunoreactivity, the underlying white matter showed only weak staining, explaining the low band intensity for the cerebellar region as a whole.

### Antibody specificity

**Figure 2:**
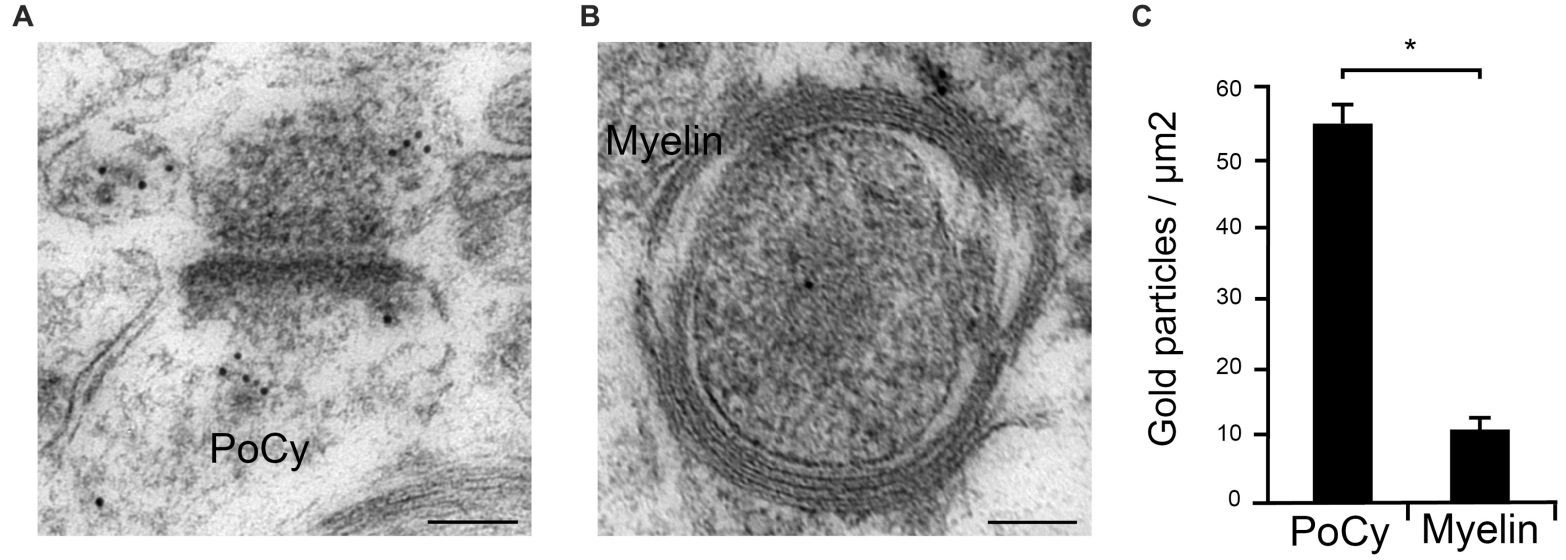
Quantitative analysis of Arc immungold labeling in the postsynaptic cytoplasm and myelin profiles. **(A)** Postsynaptic cytoplasm (PoCy) of hippocampal synapses consistently shows Arc immunogold labeling under the electron microscope. **(B)** In contrast, myelin sheaths showed virtually no Arc labeling. **(C)** Quantification (*N* = 291) of immunogold labeling showed significant (*p* < 0.0001) higher concentration of Arc in the postsynaptic cytoplasm (PoCy) compared to the myelin sheaths.

The specificity of the Arc antibody has previously been verified by immunocytochemistry of dissociated hippocampal neuron cultures prepared from WT and Arc KO littermates (Niere et al., 2012), and we have also characterized it in a recent study (Egbenya et al., 2023). However, considerable effort was employed to verify the specificity of the anti-Arc antibody used in our quantitative immunogold electron microscopical studies. As a negative control, we quantitated the immunogold labeling over myelin sheaths around axons and compared to levels in the postsynaptic cytoplasm (Figure 2). The postsynaptic cytoplasm has a well-established expression of Arc, as shown consistently in our electron micrographs (Figure 2A). However, the myelin sheaths were virtually devoid of immunogold particles (Figure 2B), though the axon profiles inside the sheaths were typically labeled, in spite of the fact that these sheaths contain high concentrations of other “sticky” proteins (Morell, 1999). Besides neurons, Arc expression has also been shown in astrocytes, but not in oligodendrocytes (Rodriguez et al., 2005; Schochet et al., 2005; Uhlen et al., 2015). We chose here to use the myelin sheaths as *bona fide* knock-out tissue, with the additional advantage that this tissue had been subject to the exact same fixation and other treatments as the synaptic structures investigated. Quantitative analyses of synaptic and myelin profiles revealed a highly significant difference in gold particle densities between the postsynaptic cytoplasm (55.2 gold particles/μm^2^ ± 3.7) and the myelin sheaths (10.4 gold particles/μm^2^ ± 2.2) (*n* = 291 in the PoCy group, *n* = 56 in the myelin group, *p* < 0.0001, Mann–Whitney U test) (Figure 2C). As additional control measurements, sections were incubated with primary antibody coupled with subsequent incubation with a secondary antibody from an inappropriate species, revealing no labeling at all. Also, no labeling was observed when sections were incubated in secondary antibody only, omitting primary antibody incubation. We concluded that the Arc antibody is specific when used under the present electron microscopical conditions.

### Arc immunogold labeling of excitatory synapses

**Figure 3:**
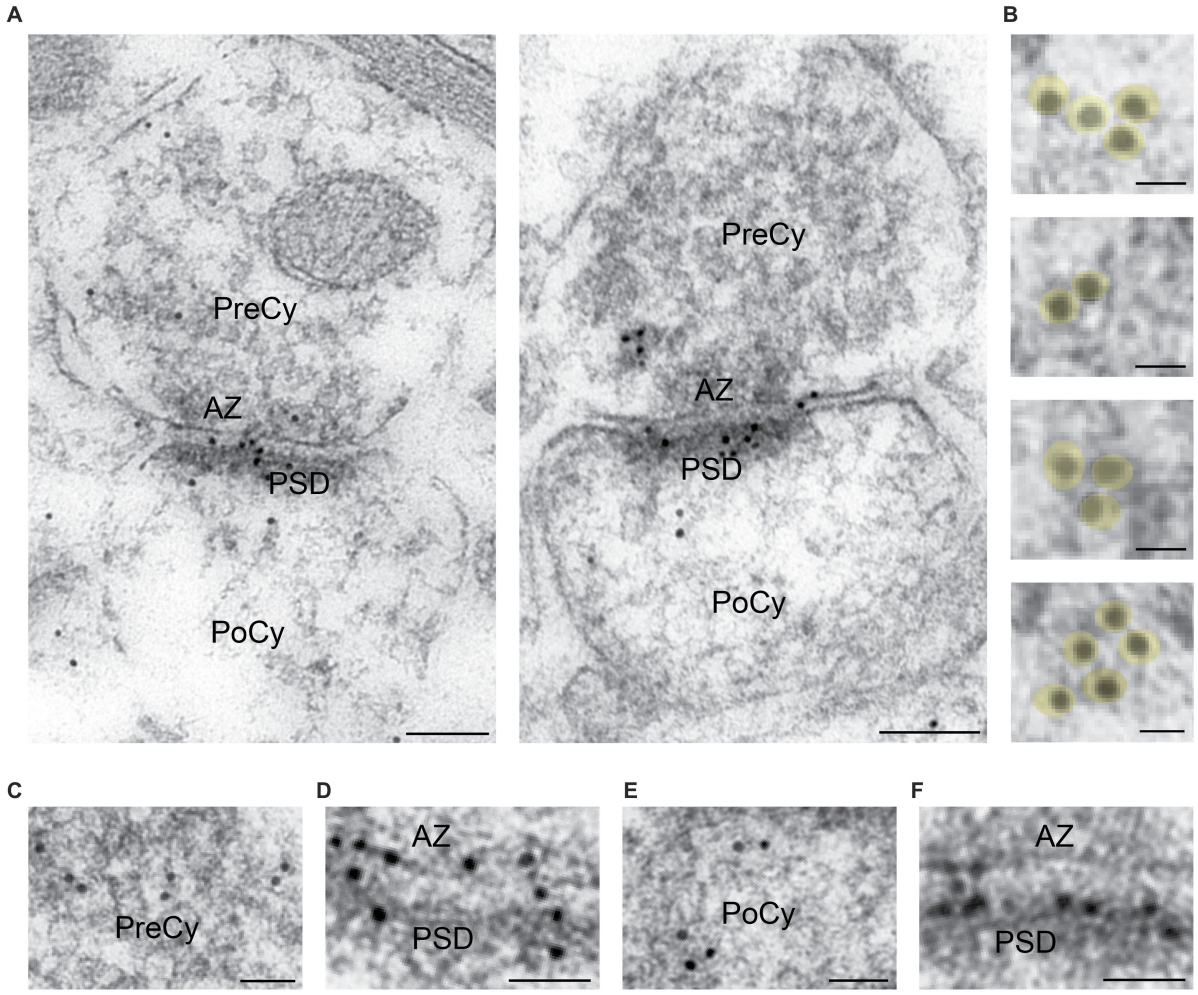
Electron microscopy of Arc immunogold labeling in hippocampal asymmetric synapses from the CA1 region of the rat hippocampus. **(A)** Electron micrographs of Arc immunogold labeling of excitatory synapses. **(B)** Arc labeling often appeared as clusters of gold particles over vesicles. **(C)** Arc labeling of the presynaptic cytoplasm at higher magnification. **(D)** Arc labeling of active zone at higher magnification. **(E)** Arc labeling of postsynaptic cytoplasm at higher magnification. **(F)** Arc labeling of PSD at higher magnification. PreCy, presynaptic cytoplasm; AZ, active zone; PoCy, postsynaptic cytoplasm; PSD, Postsynaptic density. Scale bars: **(A)** 100 nm, **(B)** 20 nm, **(C–F)** 50 nm.

**Figure 4:**
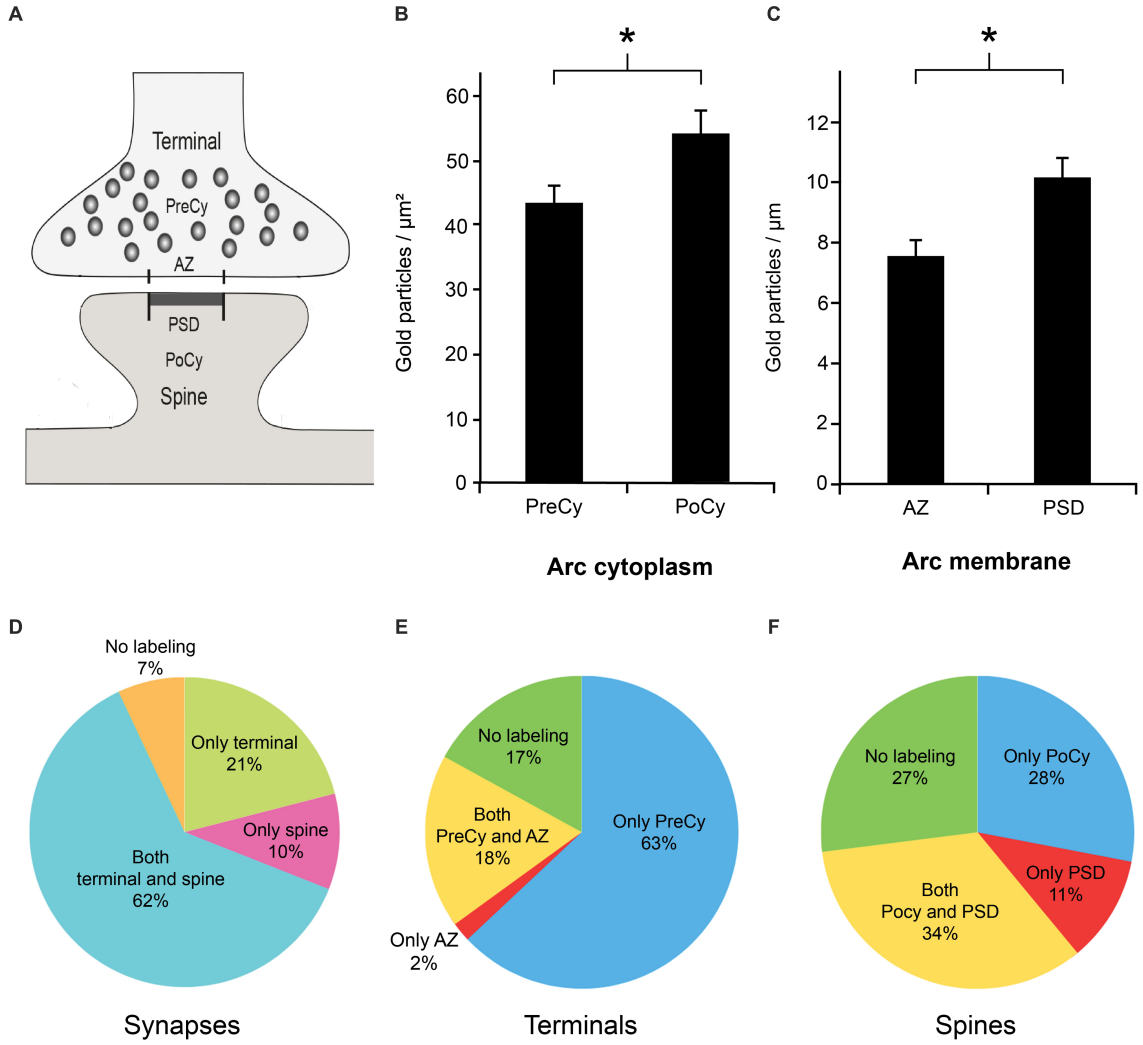
Quantitative analysis of Arc immunogold labeling in the subregions of the synapse. **(A)** Schematic illustration of synaptic regions used for the quantitative analysis. The presynaptic cytoplasm (PreCy); the active zone (AZ); the postsynaptic cytoplasm (PoCy); the postsynaptic density (PSD). **(B)** Mean Arc immunogold labeling density over cytoplasmic regions of interest. **(C)** Mean Arc immunogold labeling density over plasma membrane regions of interest. **(D)** Percentage of Arc immunogold labeling of synaptic regions. **(E)** Percentage of Arc immunogold labeling in subregions of presynaptic terminal. **(F)** Percentage of Arc immunogold labeling in subregions of postsynaptic spine. Asterisks denote statistically significant difference (*p*-value cytoplasm: < 0.05, *p*-value membrane: < 0.01). Error bars denote SEM.

We found that both presynaptic terminals and postsynaptic spines in Schaffer collateral synapses in the stratum radiatum of the CA1 region in the rat hippocampus displayed Arc immunogold labeling (Figure 3A). The labeling of Arc often appeared as clusters of gold particles associated with about 2–5 vesicles in the cytoplasmic regions of interest (Figure 3B). The presynaptic vesicles were about the size of regular synaptic vesicles, i.e., about 40 nm in diameter. In the presynaptic cytoplasm, the immunogold particles were present over some synaptic vesicles (Figure 3C), i.e., the gold particles were localized either close to the vesicle membrane or within the vesicle lumen. The protein was also present over the plasma membrane, predominantly over the active zone (Figure 3D). In the postsynaptic spine, several gold particles were seen in the postsynaptic cytoplasm (Figure 3E). The highest density of Arc in the spines was typically observed over the postsynaptic density (PSD) (Figure 3F). Some vesicles in or close to the PSD were Arc positive. Quantitative analysis of Arc immunogold labeling density in synaptic ROIs (Figure 4A) significantly showed 25% higher concentration of the protein in the postsynaptic cytoplasm (55.2 gold particles/μm^2^ ± 3.7) compared to the presynaptic cytoplasm (44.0 gold particles/μm^2^ ± 2.9) (*n* = 291 in both groups, *p* = 0.029, Mann–Whitney U test) (Figures 4B). Similarly, the concentration of Arc in the PSD (10.3 gold particles/μm ± 0.7) was significantly 26% higher than in the presynaptic active zone (7.6 gold particles/μm ± 0.6) (*n* = 291 in both groups, *p* = 0.006, Mann–Whitney U test) (Figure 4C). Quantification of Arc immunogold labeling of synapses revealed that Arc is expressed exclusively in the presynaptic terminal in 21% of synapses and solely in the postsynaptic spine in 10% of synapses. The majority of synapses (62%) exhibited simultaneous presence of Arc in both the terminal and the spine. Approximately 7% of synapses showed no immunoreactivity for Arc (Figure 4D). Quantitative analysis of Arc immunogold labeling in the presynaptic terminals revealed that 63% of terminals displayed Arc immunogold labeling exclusively in the presynaptic cytoplasm, while 2% exhibited labeling specifically at the active zone. In 18% of terminals, Arc was localized in both the presynaptic cytoplasm and at the active zone, whereas 17% of terminals showed no immunoreactivity for the protein (Figure 4E). Furthermore, within each synaptic terminal, there was an average presence of approximately 40 synaptic vesicles. Among these presynaptic vesicles, a fraction of 6% exhibited the presence of Arc protein. Quantification of immunogold labeling in the postsynaptic spines demonstrated that Arc is present particularly in the postsynaptic cytoplasm in 56% of spines, while 3% showed specific labeling at the postsynaptic density. Furthermore, in 14% of spines, Arc was localized in both the postsynaptic cytoplasm and at the postsynaptic density. However, 27% of spines displayed no immunoreactivity for Arc protein at all (Figure 4F).

### Arc in extracellular vesicles

**Figure 5:**
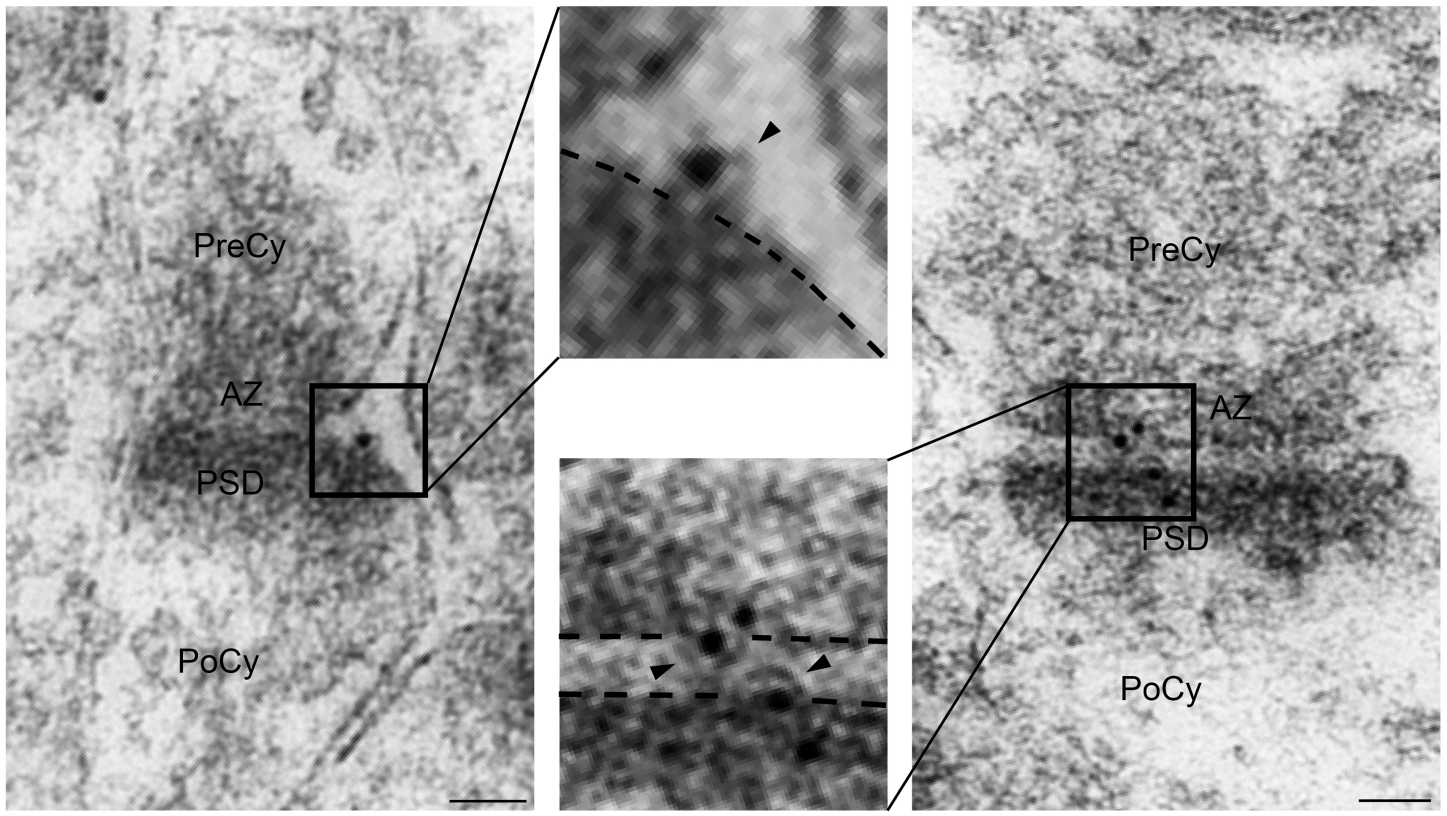
Electron micrographs displaying Arc positive extracellular vesicles close to the synapse or in the synaptic cleft. The extracellular vesicles (arrowheads) are shown at higher magnification in insets. PreCy, presynaptic cytoplasm; AZ, active zone; PoCy, postsynaptic cytoplasm; PSD, Postsynaptic density. Scale bars: 50 nm.

**Figure 6:**
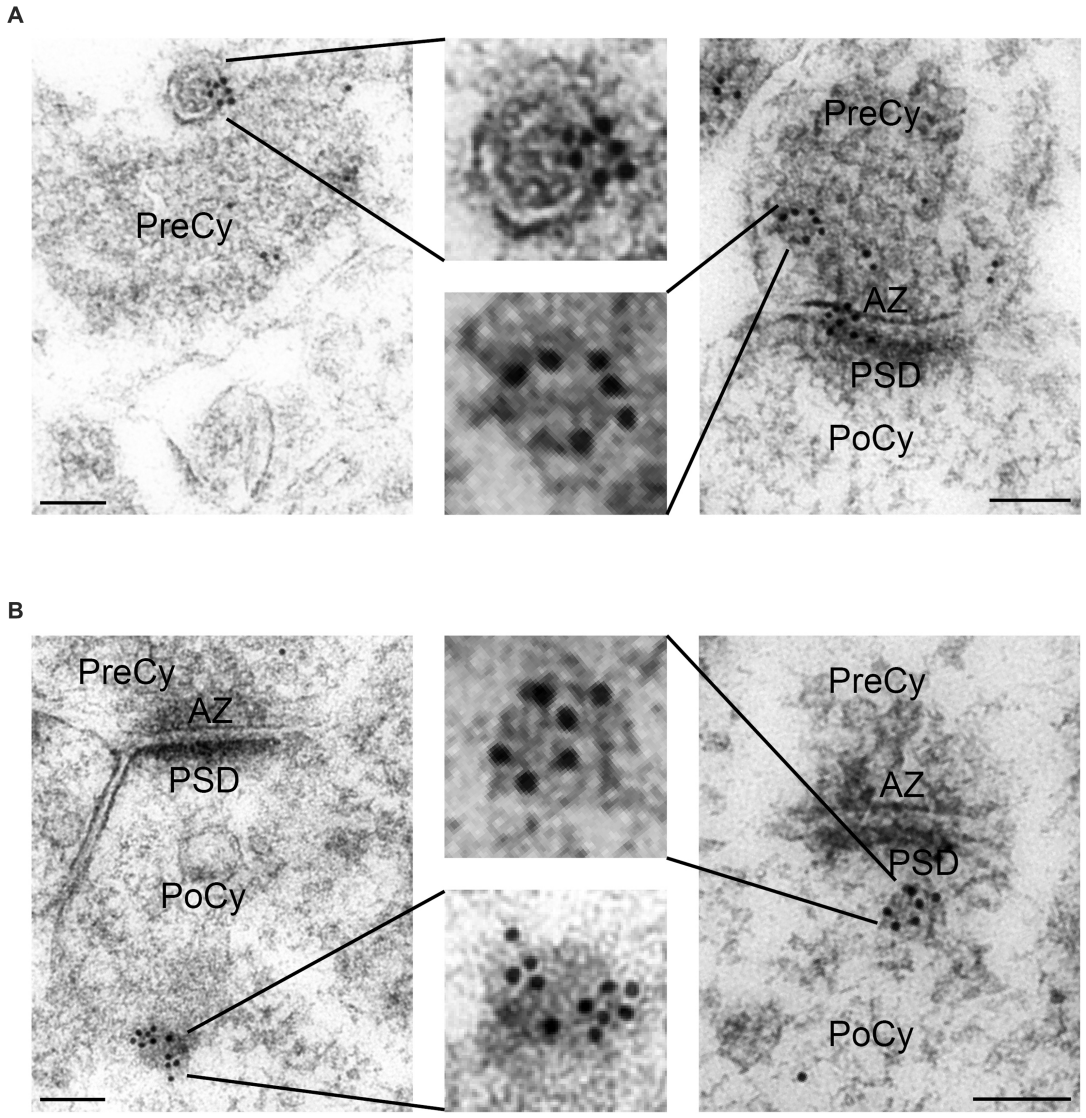
Electron micrographs displaying synaptic Arc positive multi vesicular body like structures (MVBLS). **(A)** Arc positive MVBLS in the presynaptic terminals. **(B)** Arc positive MVBLS in the postsynaptic spines. The MVBLS are shown at higher magnification in insets. PreCy, presynaptic cytoplasm; AZ, active zone; PoCy, postsynaptic cytoplasm; PSD, Postsynaptic density. Scale bars: 100 nm.

Extracellular vesicles (EVs) with an approximate diameter of 26 nm were detected in the synaptic cleft or close to the synapse (Figure 5). Some of these vesicles were labeled for Arc. Unlike the clusters of gold particles associated with synaptic vesicles in the cytoplasm, only single gold particles were observed attached to these extrasynaptic vesicles. EVs were detected in 10% of the synapses. Approximately 6% of synapses showed Arc-positive EVs, while 4% of synapses exhibited Arc-negative EVs. Overall, 63% of the synaptic EVs contained Arc, while the remaining 37% were immunonegative for the protein (Table 1). We also found examples of clusters of vesicles, some of which were enclosed by an outer membrane within the cytoplasm. These round structures resembled multivesicular bodies and were classified as multivesicular body-like structures (MVBLS). Some of these MVBLS were labeled with several gold particles (Figure 6). The MVBLS were observed both in the presynaptic terminals (Figure 6A) and postsynaptic spines (Figure 6B). Approximately 4% of presynaptic terminals contained MVBLS, while 5% of postsynaptic spines displayed MVBLS. Interestingly, within both the presynaptic terminals and the postsynaptic spines, there was an equal distribution, with 50% of the MVBLS being positive for Arc and the remaining 50% being negative for Arc (Table 2).

**Table 1:** The table shows electron microscopical quantitative analysis of extracellular vesicles in hippocampal synapses.

**Synapses without extracellular vesicles**	171/190	90%
**Synapses with extracellular vesicles**	19/190	10%
Synapses with Arc postive extracellular vesicles	12/190	6.3%
Synapses with Arc negative extracellular vesicles	7/190	3.7%
- Arc positive extracellular vesicles	12/19	63%
- Arc negative extracellular vesicles	7/19	37%

**Table 2:** The table shows electron microscopical quantitative analysis of multivesicular body like structures (MVBLS) in hippocampal synapses.

**Synapses without MVBLS**	172/190	90%
**Synapses with MVBLS**	18/190	10%
**Synapses with MVBLS in the terminal**	8/190	4.2%
Synapses with Arc positive MVBLS in the terminal	4/190	2.1%
Synapses with Arc negative MVBLS in the terminal	4/190	2.1%
- Arc positive MVBLS in the terminals	4/8	50%
- Arc negative MVBLS in the terminals	4/8	50%
**Synapses with MVBLS in the spine**	10/190	5.2%
Synapses with Arc positive MVBLS in the spine	5/190	2.6%
Synapses with Arc negative MVBLS in the spine	5/190	2.6%
- Arc positive MVBLS in the spines	5/10	50%
- Arc negative MVBLS in the spines	5/10	50%

### Arc in dendrites and axons

**Figure 7:**
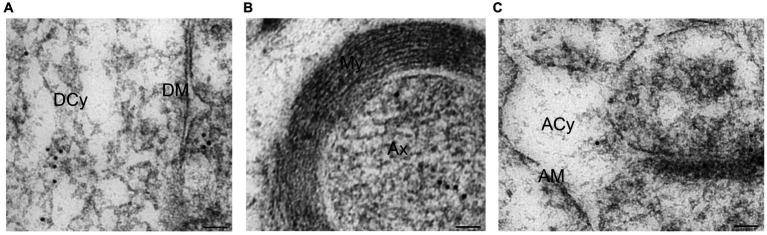
Electron micrographs displaying Arc immunogold labeling in dendrite, axon and astrocyte. **(A)** Arc immunogold labeling in dendrite. **(B)** Arc immunogold labeling of a myelinated axon. **(C)** Arc immunogold labeling in an astrocyte. Abbreviations: DCy, dendritic cytoplasm; DM, dendritic membrane; My, myelin; Ax, axon; ACy, astrocytic cytoplasm; AM, astrocytic membrane. Scale bars: 50 nm.

In the dendrites immediately adjacent to the spines, Arc was present in the cytoplasm attached to single vesicles (Figure 7A) or MVBLS. Labeling of dendritic membrane was rarely observed. Arc was clearly present also in the axonal cytoplasm (Figure 7B). The protein was not, however, observed in myelin sheaths surrounding the axons. Occasionally, isolated gold particles were noted within the cytoplasm of astrocytes (Figure 7C). This observation was interpreted as incidental background labeling. Notably, Arc was not detected at the astrocytic membrane.

## Discussion

We have investigated the ultrastructural localization of Arc protein at glutamatergic synapses in the rat hippocampus using semi-quantitative postembedding immunogold electron microscopy. Interestingly, Arc labeling was observed in both the pre-and postsynaptic cytoplasm, most often associated with clusters of small vesicles, as well as along the plasma membranes, most predominantly along the PSD. Notably, we also found extracellular vesicles in the synaptic cleft that were labeled for Arc.

The presence of Arc in hippocampal presynaptic terminals has not yet been firmly established. Some previous studies have observed that Arc labeling has not been consistently observed in presynaptic compartments in some brain regions (Fujimoto et al., 2004; Rodriguez et al., 2005). However, others have found that the Drosophila Arc homologue, dArc1, associates with its own transcript and is transported from presynaptic boutons in neuromuscular junctions to postsynaptic sites, likely through extracellular vesicles (Ashley et al., 2018). Our study suggests, however, that Arc can be found also at presynaptic sites in the mammalian CNS, here in the hippocampal Schaffer collaterals in stratum radiatum. We have recently also shown that the synaptic concentrations of Arc (and BDNF) may change in chronic temporal lobe epilepsy, compared to normal controls (Egbenya et al., 2023).

In line with previous morphological description of exosomes (Fevrier and Raposo, 2004; Conde-Vancells et al., 2008), we observed extracellular vesicles of about 26 nm in diameter in and close to the synaptic cleft. These vesicles were labeled for Arc. According to previous studies, Arc may be released from neurons in extracellular vesicles, thus mediating the intercellular transfer of proteins and mRNA in cells such as HEK293 (Pastuzyn et al., 2018). As mentioned earlier, Ashley and coworkers have reported some evidence to support the hypothesis that Arc may be transferred from presynaptic compartments to postsynaptic sites, in a retroviral-like mechanism (Ashley et al., 2018). Multivesicular bodies can potentially release their content into the extracellular space upon fusion with the plasma membrane, giving rise to small extracellular vesicles in the synaptic cleft (Nieves Torres and Lee, 2023), as observed in this study.

Though our observations support the concept of transsynaptic signaling with Arc-containing vesicles, our results alone do not give any information about the direction of such signaling. We find Arc-positive vesicles and multi-vesicular-like bodies in both presynaptic terminals, and postsynaptic spines. In principle, this signaling could go both ways.

Genetic information could possibly be transferred between neurons through this molecular mechanism of transsynaptic signaling (Pastuzyn et al., 2018). Some studies suggest that such intercellular signaling in the nervous system may in fact be mediated by extracellular vesicles, modulating the properties and connectivity of synapses, as well as contributing to synaptic plasticity (Budnik et al., 2016; Zappulli et al., 2016; Ashley et al., 2018).

Furthermore, Arc-positive cytoplasmic vesicles were often found close to the plasma membrane. This is in line with the proposed mechanism of exocytosis of Arc-containing vesicles or multivesicular bodies with the plasma membrane (Thery et al., 2002). Our localization of Arc in cytoplasmic vesicles close to the synaptic plasma membrane, as well as in extracellular vesicles in the synaptic cleft, further support the idea that Arc-positive extracellular vesicles may be involved in the trafficking of Arc across synapses. Others have also supported the view that exosomes may mediate intercellular communication between cortical neurons, not just in the neuromuscular synapse (Chivet et al., 2014). In fact, extracellular vesicles containing Arc in the form of virus-like capsids have been harvested from cultured mouse cortical neurons (Pastuzyn et al., 2018). Also, Drosophila Arc1-capsid binds dArc1 mRNA in neurons before it is loaded in extracellular vesicles and transferred from motor neurons to muscle cells (Ashley et al., 2018). These capsids and their surrounding vesicles were found to have a mean diameter of about 30 nm. Erlendsson et al. (2020), however, used cryo-electron microscopy to generate a high-resolution map of the bacterially expressed Drosophila Arc capsid, which they found had a mean diameter of 37 nm. Eriksen et al. (2021), on the other hand, found that capsid-like structures from human WT Arc expressed in *E. coli* have a diameter of ~30 nm. Though these diameters are means calculated from a range of independent observations, our observed mean diameter of about 26 nm is somewhat smaller than in these other studies. This discrepancy, which may be based on technical issues, is still small enough to make it likely that we are looking at the same type of Arc-containing vesicles that have been observed in these other studies. The small size of these vesicles, as noted by us and others, may indicate that they are exosomes (30–150 nm in diameter), which are typically released from multivesicular bodies, but we cannot exclude that they (or some of them) are microvesicles which are formed by outward budding of the plasma membrane, though these are typically somewhat larger than in our material (from 100 nm) (Doyle and Wang, 2019; Cano et al., 2023).

If some of the vesicle-like profiles observed in the synaptic cleft in fact are budding or fusing extracellular vesicles, not “free” vesicles, it is important to note that (1) the synaptic cleft is so narrow (20–30 nm) that such extracellular vesicles will inevitably touch the membranes if they are located within the cleft and (2) if extracellular vesicles are not located adjacent to or touching a plasma membrane, they may not be detected at all, they will more likely have been washed away during perfusion and fixation of the brain.

The accumulation of Arc along the PSD may be a result of its contribution to the endocytosis of AMPA receptors, which are also highly concentrated at the PSD in these synapses (Feligioni et al., 2006). Arc is previously demonstrated to interact with endophilin and dynamin to increase AMPA receptor endocytosis, and thus reducing its membrane expression (Chowdhury et al., 2006). Interestingly, Arc protein may on the one hand associate with brain liposomes through palmitoylation (Barylko et al., 2018), but it may also interact directly with PSD95 in the synapse (Fernandez et al., 2017), recruiting it to excitatory synapses in an indirect manner.

Caution should be made, however, due to the fact that our observations only represent snapshots in the life of the Arc-positive vesicles. Though our results may be in line with multivesicular bodies being a source of Arc-containing extracellular vesicles, it may also be the other way round, that what we interpret as multivesicular bodies may be endocytotic organelles. As noted above, we cannot conclusively tell whether the extracellular vesicles we have observed originate from the presynaptic or postsynaptic compartments, i.e., in which direction they are sent. It would be interesting to harvest extracellular vesicles from our hippocampal material, in order to investigate if vesicles obtained in that way also contain Arc.

Our observations do not constitute a final conclusion regarding subsynaptic localizations of Arc, but they seem to support some interesting functional principles, not least its presynaptic localization in addition to the postsynaptic, and the possible existence of Arc-positive extracellular vesicles. Further research is needed to confirm and elaborate on these principles, not least using KO animals as controls. KO control of the same antibody as used by us has, in fact, been performed in another study (Niere et al., 2012). In the present study, however, we have compared synaptic immunogold labeling with labeling over myelin sheaths, which do not contain Arc protein. The advantage of supplementing with the myelin control is that it is performed in the same species (rat), even in the very same animals, and the same brain area (hippocampus), as the synapses under investigation. In future studies, it may also be an option to do an alternative test of immunolabeling specificity by performing a simulation where one randomly distributes the same overall density of particles over the section images and then quantify how often the regions of interest are simulated as Arc-positive. The experimental results may then be compared with the randomized simulation.

## Conclusion

Our main results, based on quantitative immunogold analyses, are that Arc is expressed in both pre-and postsynaptic terminals in the hippocampus, with highest levels in the postsynaptic spines. Accumulation of Arc along the PSD is in line with the notion that it contributes to endocytosis of AMPA receptor at this site. Arc was also observed in extracellular vesicles, as well as in multivesicular bodies, which may support the hypothesis that Arc may be transferred between pre-and postsynaptic sites as part of a transsynaptic signaling mechanism.

## Data availability statement

The raw data supporting the conclusions of this article will be made available by the authors, without undue reservation.

## Ethics statement

The animal study was approved by Institutional Animal Care and Use Committee at University of Oslo. The study was conducted in accordance with the local legislation and institutional requirements.

## Author contributions

SH and SD: conceived and designed the experiments. HR, DLE, and MB: electron microscopy experiments, quantification of the EM data, and analyzed the data. HR: light microscopy experiments. DLE: western blot experiments. SH, SD, HR, DLE, and MB: interpretation of the data. SH, SD, and HR: drafting the article and critical revision of the article. All authors contributed to the article and approved the submitted version.
